# Efficacy of Vermiremediation to Remove Contaminants from Soil

**DOI:** 10.5696/2156-9614-11.29.210302

**Published:** 2021-02-25

**Authors:** Ebenezer Olasunkanmi Dada, Modupe Olatunde Akinola, Stephen Olugbemiga Owa, Gabriel Adewunmi Dedeke, Adeyinka A. Aladesida, Folarin O. Owagboriaye, Emmanuel O. Oludipe

**Affiliations:** 1 Department of Cell Biology and Genetics, Environmental Biology Unit, Faculty of Science, University of Lagos, Akoka, Yaba, Lagos, Nigeria.; 2 Applied Biology and Biotechnology Programme, Landmark University, Omu-Aran, Nigeria.; 3 Department of Pure and Applied Zoology, College of Bioscience, Federal University of Agriculture, Abeokuta, Nigeria.; 4 Department of Zoology and Environmental Biology, Faculty of Science, Olabisi Onabanjo University, Ago Iwoye, Nigeria.

**Keywords:** contamination, metallothioneins, pollutants, pollution, remediation

## Abstract

**Background.:**

In addition to improving soil fertility and crop production, earthworms have been found to be useful in the removal of contaminants from soil, known as vermiremediation. Previous studies on vermiremediation have focused primarily on organic wastes, with relatively less attention paid to inorganic contaminants. In addition, some basic terms used in environmental health studies have often not been properly clarified.

**Objectives.:**

The present study is a review of the state of the literature on the effectiveness of using earthworms to remediate organic and inorganic (metal) soil contaminants. Earthworms’ actions in remediation of organic and inorganic contaminants are described. Some terms that are used interchangeably in environmental health are clarified. The challenges and limitations of vermiremediation are highlighted.

**Methods.:**

A systematic literature search was conducted to access online academic publications indexed in Google Scholar, PubMed, Scopus, Clarivate Analytics (Web of Science), ScienceDirect, ResearchGate and Springer Link. A total of 165 publications on the subject matter were accessed, out of which 47 were used for the review.

**Discussion.:**

Empirical and theoretical information from the literature showed evidence of the significant contributions of earthworms to the removal of soil organic contaminants and metals. Earthworms indirectly facilitate the conversion of organic contaminants by promoting microbial and enzyme activities. Some organic contaminants are directly taken up through dermal and intestinal absorption and accumulated by preferential sequestration in sub-organismic and tissue fractions of earthworms. Metals are directly removed and accumulated by the mechanism of detoxification and sequestration, via metallothioneins induction. The terms ‘contaminants’ and ‘pollutants’ have different meanings and should not be used interchangeably. Although vermiremediation presents an ideal clean-up technique, it is limited in application to only mildly contaminated soil environments. Ethical concerns should not pose a serious issue because vermiremediation simply takes advantage of earthworms’ natural soil-conditioning abilities. Many vermiremediation processes, especially of organic wastes, are harmless to earthworms, improving the soil for their growth and survival.

**Conclusions.:**

Vermiremediation presents a good long-term biological option to clean up mildly contaminated soil. It may be deployed as a secondary measure to rid the soil of residual contaminants after applying physicochemical remediation techniques to an overtly polluted soil environment.

**Competing Interests.:**

The authors declare no competing financial interests.

## Introduction

Earthworms promote plant growth and productivity through complex mechanical and biochemical interactions with soil abiotic and biotic components.^1^ As earthworms burrow, they ingest soil; this results in mechanical breakdown of soil particles, and increased surface areas for biotic actions. Earthworm burrows act as pathways for water movement, particle movement, nutrient flow, and aeration. The guts of earthworms are host to millions of enzymes and microorganisms which facilitate the rapid biochemical conversion and mineralization of soil organic matters, thereby enriching soil.^1^ All these processes, together with other factors, facilitate increased plant growth and crop yield.

It is recognized that earthworms also have the potential to mitigate many environmental challenges. The science of using earthworms to improve food production and tackle environmental and other human challenges is referred to as vermitechnology.^1,2,3^ An important aspect of vermitechnology that has received relatively less attention is vermiremediation, a term used to describe the process by which earthworms clean up soil contaminants. Vermiremediation utilizes earthworms’ biotic and abiotic interactions, life cycle, burrowing and feeding behavior to transform, degrade, or remove contaminants from the soil environment.^4^

Globally, soil is subjected to anthropogenic pollution and contamination from industrial, farming, and other activities. Soil contaminants typically include chemicals, organic wastes, inorganic compounds or elements, especially metals.^2^ Due to high costs and ecological and environmental destabilization associated with traditional physicochemical remediation methods, attention is now shifting to biological *in situ* alternatives. Some research and review studies on vermiremediation are available, but most of these focus on organic contaminants. In addition, insufficient attention has been paid to the mechanisms of actions of earthworms in vermiremediation of inorganic contaminants, especially metals. Some basic terms used in environmental health matters have also not been properly clarified. This review aimed to present the state of the literature on the effectiveness of using earthworms to remediate organic and inorganic (metals) soil contaminants. The mechanisms of action of earthworms in the remediation of both soil organic and metal contaminants are described. The review also seeks to clarify some terms that are sometimes used interchangeably in environmental and remediation matters, including contaminants, pollutants, contamination, and pollution.

**Abbreviations**
*MT*: Metallothionein
*PAH*: Polycyclic aromatic hydrocarbon

## Methods

**Figure 1:**
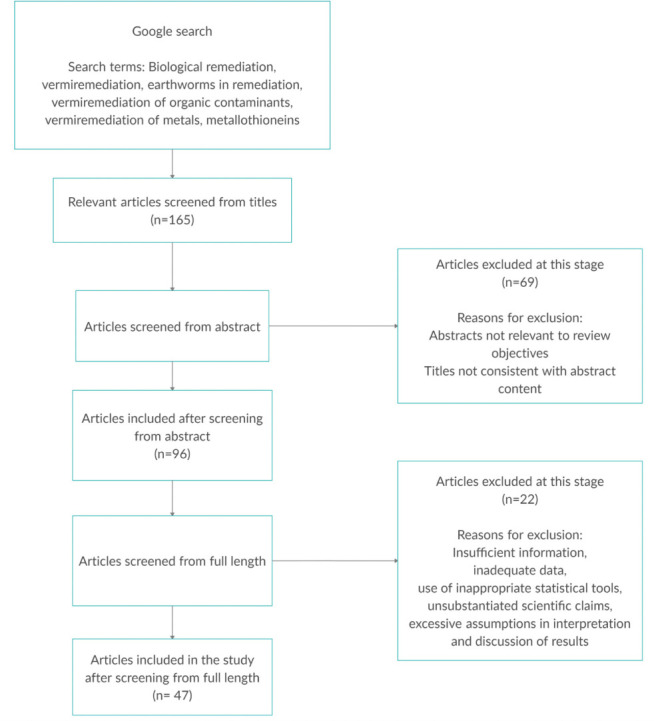
PRISMA flow chart showing literature collection, screening, inclusion, and exclusion procedure

A systematic literature search was conducted using the Google search engine to access online academic publications indexed in Google Scholar, PubMed, Scopus, Clarivate Analytics (Web of Science), ScienceDirect, ResearchGate and Springer Link. The words, terms, clauses, and phrases used in the literature search included biological remediation, bioremediation, vermiremediation, use of earthworms in remediation, vermiremediation of organic contaminants, vermiremediation of inorganic contaminants, vermiremediation of metals, pollution and contamination, pollutants and contaminants, and metallothioneins. A total of 165 peer reviewed publications were accessed based on the relevance of titles to the study. These were further screened to 96 after reading through their abstracts. After screening the full length of the papers, 47 were used for this review, excluding the Preferred Reporting Items for Systematic Reviews and Meta-Analyses (PRISMA) reference.^5^ Articles were excluded as a result of irrelevance of abstracts to our review objectives, inconsistencies of titles with abstract and full length content, inadequate data presentation, use of inappropriate statistical tools, unsubstantiated scientific claims, or excessive assumptions in interpretation and discussion of results. The last day of article search was July 30, 2020. Literature collection, screening, inclusion, and exclusion procedures are shown in Figure 1.

## Results

Of the 47 academic articles included in the present review, only four attempted to define or clarify some basic terms used in environmental health - ‘pollution’, ‘pollutants’, ‘contamination’, and ‘contaminants’.^2,6,7,8^ Information gathered from the literature indicates that each of the two pairs of terms: ‘contaminants’ and ‘pollutants’ and their respective derivatives, ‘contamination’ and pollution’, are related, but technically, they have different meanings.^2,6,7,8^ The term ‘contaminant’ refers to any substance (liquid, solid, or gaseous) that although may be inherently or potentially harmful, at its present quantity, concentration or volume, cannot cause any harm or adverse effects to the environment where it is present, or to the resident organisms.^2,6,7,8^ Hence, a potentially harmful substance may not be referred to as a ‘pollutant’ if its concentration, volume, or quantity is not enough to cause harm. But when the quantity, concentration, or volume of the substance build up beyond tolerable limits, such that it is now causing harm or adverse effects, it then becomes a pollutant, and is referred to as such.^2,6,7,8^

Contamination refers to the presence of any substance in the environment, that though it may be intrinsically harmful, but at the quantity, concentration, or volume that it is currently present, it is not causing any harm or adverse biological effects.

Pollution on the other hand, refers to the presence of any substance in the environment that is harmful or causing adverse biological effects to the environment or resident organisms.^2,6^

Unlike the physical and chemical remediation techniques which are the first, and in many cases, the only line of action in combating cases of overtly high pollution, biological remediation methods that employ living organisms such as plants, microorganisms, and animals, like earthworms, can only be applied to mildly contaminated soil or water.^2,7,8^ Hence, in bioremediation, including vermiremediation, the terms contaminants, contamination and contaminated soil are more appropriate. Table 1 gives further clarifications of these terms.

**Table 1:** Characteristics of Contaminants and Pollutants in Contrast to Contamination and Pollution

	**Contaminants**	**Pollutants**
**State of matter of the substance**	May be solid, liquid, or gas	May be solid, liquid, or gas
**Nature of substance**	May be inherently/potentially harmful	May be inherently/potentially harmful
**Volume, concentration, or quantity in its environment**	In traces/relatively low/below permissible/tolerable levels	Above tolerable/permissible level. Relatively high concentration, volume, or quantity. Toxic chemicals or substances may be harmful even in traces or low concentrations.
**Current effects on the environment/ecological system/living organisms**	Not harmful. Not causing harmful/adverse reactions or effects.	Harmful. Causing harmful/adverse reactions or effects.
**How applicable to vermiremediation?**	Applicable	Usually not applicable
**Example**	Organic/inorganic compounds in the soil of a farmland, a body of water, or the atmospheric air, in harmless concentrations or below permissible/tolerable levels	Organic/inorganic compounds in the soil of a farmland, a body of water, or the atmospheric air, in harmful concentrations, or above permissible/tolerable levels

	**Contamination**	**Pollution**

**State of the environment**	Presence of substances in the environment in concentrations, volumes, or quantity that are not harmful.	Presence of substances in the environment in concentrations, volumes, or quantity that are harmful.
**How applicable to vermiremediation?**	Applicable	Usually not applicable
**Example**	Presence of organic/inorganic compounds in the soil of a farmland, body of water, or atmospheric air, in harmless concentrations or below permissible/tolerable levels.	Presence of organic/inorganic compounds in the soil of a farmland, body of water, or atmospheric air, in harmful concentrations, or above permissible/tolerable levels.

### Vermiremediation characteristics

All the accessed articles related to the use of earthworms as clean-up agents were unanimous in their definition of vermiremediation as the use of earthworms to clean up contaminated sites, usually soil. This implies agreement with the finding that some earthworms are tolerant to, and can remove, or aid the removal of a number of organic and inorganic contaminants from soil. Organic contaminants may include crude oil, chemicals, pesticides, and polycyclic aromatic hydrocarbons (PAHs). Inorganic contaminants may include metals, such as cadmium (Cd), lead (Pb), mercury (Hg), and arsenic (As).^1,2,9,10^

Articles that reported results of field or laboratory vermiremediation experiments either worked on organic contaminants or inorganic contaminants. There were seven articles that reported vermiremediation of organic wastes or contaminants.^11,12,13,14,15,16,17^

### Remediation of organic contaminants by earthworms

Organic matters or substrates are generally known to be biodegradable through a biotransformation process known as mineralization or composting. When earthworms facilitate or speed up the process of natural composting of organic matter, it is referred to as vermicomposting. Vermicomposting is the process of biodegradation of organic matter through the interactions between earthworms and microorganisms. When the organic matter or substrate is a contaminant, the earthworm-facilitated composting is described as vermiremediation. An overview of each published academic article that reported evidence of vermiremediation of organic contaminants is presented in the following paragraphs and Table 2.

**Table 2:** Studies on Vermiremediation of Organic Contaminants

**Study**	**Substrate**	**Inorganic contaminant**	**Earthworm species/earthworm product**	**Duration**	**Effectiveness of remediation**
Almutairi (2019)^11^	Soil	Petroleum hydrocarbons (TPH)	*E. fetida, L. terrestris*	60 days	*L. terrestris* reduced TPH by 28.0%; *E. fetida* reduced TPH by 33.0%; *E. fetida* and *L. terrestris* jointly reduced TPH by 35.0%
Chachina *et al.* 2016^12^	Soil	Petroleum and diesel oil	*E. fetida* with and without activation biopreparation	22 weeks	*E. fetida* aided petroleum oil degradation (99%). Biopreparation had no significant effect. Remediation of diesel oil not successful
Ahmed *et al.* 2020^13^	Soil	Chlorpyrifos insecticide	Earthworm species not clearly indicated	3–45 days	Earthworms aided degradation of up to 64.3 – 66.5%
Rorat *et al.* 2017^14^	Sewage sludge	PAHs	*E.* a*ndrei*	30 days	High removal of PAHs Earthworms accumulated PAHs
Chachina *et al.* 2018^15^	Soil	Petroleum and diesel oil.	*Dendrobena veneta*	22 weeks	Hydrocarbons decreased by 95% in the presence of *D. veneta*
Owagboriaye *et al.* 2019^16^	Soil	Glyphosate-based herbicide (GBH)	*Alma millsoni, Eudrilus eugeniae* and *Libyodrilus violaceus*	8 weeks	Presence of earthworms decreased glyphosate residues in the contaminated soil
Chachina *et al.* 2015^17^	Soil	Engine lubricant oil	*E. fetida, E. andrei, D. veneta*	4 months	Soil engine lubricant oil decreased by up to 99.9%

In the work of Almutairi (2019), *Eisenia fetida* and *Lumbricus terrestris* were employed to remediate soils contaminated with petroleum hydrocarbons, measured in terms of total petroleum hydrocarbons (TPHs), for a period of 60 days. At the end of the experimental period, there were significant reductions in soil TPH.^11^ In the work of Chachina *et al*. (2016),^12^ an earthworm species (*E*. *fetida*), with and without a biopreparation fluid containing bacteria, was found to remediate low hydrocarbons concentration by up to 99% after 22 weeks. Ahmed *et al*. (2020) introduced earthworms into a chlorpyrifos insecticide contaminated soil and observed significant reductions in the insecticide at the end of the 45-day experimental period.^13^

Rorat *et al*. (2017) studied the dynamics of the degradation of PAHs in the vermicomposting of sewage sludge for 30 days. The sewage sludge was mixed with bulking agents, and *Eisenia andrei* was the vermicomposting earthworm. The presence of earthworms led to a high percentage removal of PAHs.^14^ Chachina *et al*. (2018) investigated the bioremediation efficiency of a soil contaminated with oil (20 to 100 g kg^−1^), petroleum (20 to 60 g kg^−1^) and diesel fuel (20 to 40 g kg^−1^) in the presence of earthworms (*Dendrobaena veneta)* and a bio-activator preparation containing bacteria. In the oil-contaminated soil, the content of hydrocarbons decreased by 95% after 22 weeks.^15^ Owagboriaye *et al*. (2019) evaluated the biochemical response and vermiremediation potential of three tropical earthworm species (*Alma millsoni*, *Eudrilus eugeniae*, *Libyodrilus violaceus*) exposed to soil contaminated with glyphosate-based herbicide (GBH) for 8 weeks. In addition to other parameters assessed in the study, the presence of earthworms decreased glyphosate residues in the GBH soil. They concluded, in part, that both *E*. *eugeniae* and *L*. *violaceus* showed potential to vermiremediate soils contaminated with GBH.^16^ Chachina *et al*. (2015) investigated the survival and remediation potential of earthworms (*E*. *fetida*, *E*. *andrei*, *D*. *veneta*) exposed to engine lubricant oil-contaminated soil, in the presence of a bioactivator (bacteria and fungi). A remediation efficiency of 99.9% was recorded after the 4-month experimental period *(Table 2).*^17^

These studies are indications that earthworms are potentially capable of removing or reducing inorganic contaminants from the soil environment. In some of the studies, vermiremediation was effective (in terms of contaminant removal) and efficient (in term of adverse effects on earthworms) only when the contaminants were relatively low in concentrations.^11,12,14^ Hence, vermiremediation may only find good application in mildly contaminated soil environments.

#### Mechanisms of earthworm actions in remediation of organic contaminants

Most the experimental works on vermiremediation of organic contaminants reviewed in this study did not examine or report the mechanisms by which the earthworms reduced or removed the organic contaminants; the two that did, provided no details.^11,14^ Nevertheless, other articles also included in this review attempted to describe the underlying mechanisms of vermiremediation of organic contaminants, some from laboratory evidence, and others from theoretical inferences.^8,18,19,20,21,22,23,24,25,26,27,28,29^ These different strands of information are combined here to explore the mechanisms of vermiremediation of organic contaminants.

**Figure 2:**
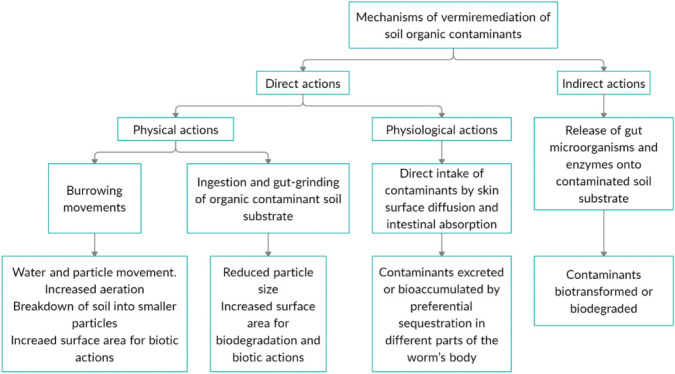
Earthworm actions in remediation of organic contaminants

Earthworms remediate organic contaminants through several interconnected processes or mechanisms that can be categorized into indirect and direct actions *(Figure 2).* Indirect biotic actions of earthworms in soil organic contaminant remediation is mainly by promoting microbial and enzyme activities in their gut and in the contaminant-bearing soil substrate.^7^ Earthworms have been found to harbor millions of biodegrader microorganisms in their gut and egest them in soil as vermicast.^18,19^ Studies have identified microorganisms associated with the gut of earthworms to include species and strains of the genera *Bacillus*, *Pseudomonas*, *Enterobacter*, *Azotobacter*, *Klebsiella*, *Proteus*, and *Streptococcus*.^18,20,21,22,23^ Studies have also shown enzymes and groups of enzymes associated with earthworm remediation activities to include amylases, proteases, lipases and cellulases.^24,25^ These microorganisms and enzymes which are deposited onto the substrates as they pass through the alimentary canal of earthworms help in facilitating the biotransformation, biodegradation and mineralization of organic matter and contaminants.

Earthworm direct actions may be physical or physiological. Earthworms impact direct physical actions by their burrowing activities. Earthworm burrows act as input points and preferred pathways for water and particle movement, and aeration. These burrowing effects result in mechanical breakdown of soil or substrate particles thereby exposing greater surface areas for biotic actions. In addition, during burrowing, earthworms ingest and digest large amounts of contaminated soils or organic matter. Digestion significantly reduces the size of the soil and contaminant particles. This also leads to increased surface area for composting microbial and enzyme actions.^1,19^

Earthworms’ direct physiological actions in organic contaminant remediation is through direct intake by passive diffusion from soil solution through the worms’ skin surface, driven by a concentration gradient between contaminants in soil pore-water and the earthworms' body fluid; and intestinal absorption of the contaminants from the soil while passing through the earthworms' gut. The contaminant so taken in is either excreted or bioaccumulated in the earthworm's body.^1,4,11,26,27^

Contaminant biotransformation in an earthworm's body is referred to as vermitransformation. In vermitransformation, degradable organic contaminants are decomposed into harmless products by enzymes (such as peroxidases) and microorganisms (bacteria and fungi) resident in the alimentary canal of earthworms and egested as in vermicomposting.^4^

Similarly, bioaccumulation of contaminants (organic or inorganic) may be referred to as ‘vermiaccumulation’ (similar to the term ‘phytoaccumulation'). Some organic contaminants are directly taken up through dermal and intestinal absorption, bioaccumulated by preferential sequestration in different parts of the earthworm's body, including the suborganismic (pre-clitellum, clitellum, post-clitellum), tissue (body wall, gut, body fluids) and subcellular (intracellular and extracellular fractions).^4,28,29^

### Remediation of inorganic contaminants by earthworms

All the studies on vermiremediation of inorganic contaminants included in the present study were on metals.^3,9,10,30,31,32,33^ Hence, in this review, metals are taken as representatives of inorganic contaminants. Metals are usually not biodegradable; hence, remediation using biological agents, especially animals like earthworms, is somewhat limited. Nevertheless, the few available studies provided strong evidence of the ability of earthworms to remediate soils mildly contaminated with metals. These studies are summarized in the following paragraphs and Table 3.

**Table 3:** Studies on the Vermiremediation of Metals

**Study**	**Substrate**	**Metals remediated**	**Earthworm/earthworm product**	**Duration**	**Effectiveness of remediation**
Marco Parra *et al.* (2010)^30^	Landfill soil, wastewater	As, Hg in soil; Ni, Cr, V, Pb in wastewater	*E. fetida* to remediate soil metals; Vermicompost used as adsorbent of wastewater metals.	60 days	As in soil reduced by 42–72%; Hg in soil reduced by 7.5–30.02%
Pattnaik and Reddy (2011)^10^	Urban waste soil	Cd, Pb, Zn, Cu, Mn	*Eudrilus eugeniae, E. fetida, Perionyx excavatus*	60 days	*E. eugeniae* reduced Pb by 32%; Zn by 37%; *E. fetida* reduced Pb by 45%; Zn by 44%; *P. excavates* reduced Pb by 51%; Zn by 56%
Shahmansouri *et al*. (2005)^9^	Biosolid	Cr, Cd, Pb, Cu, Zn	*E.fetida* from Iran; *E. fetida* from Iran Australia	2 months.	Metal concentrations in the biosolid decreased with increasing vermicomposting time
Dabke, (2013)^31^	Residual soil in excavated metal polluted soil	Cr, Pb, Cd, Fe	Resident earthworms, seeded culture of *E. fetida* earthworms, worm casts mixed with compost, photosynthetic microbial solution	2 years	Cr decreased from 192–194 mg kg^−1^ to 4.5–113.21 mg kg^−1^; Pb decrease from 5,300 mg kg^−1^ to 1,550 mg kg^−1^; No change in Cd, Fe levels
Dada *et al.* 2016^3^	Ex situ contaminated soil	*Libyodrilus violaceus*	Zn, Pb, Cd	12 weeks (72 days)	Cd reduced by 8.08 mg kg^−1^ (33.67 %); Zn reduced by 37.47 mg kg^−1^ soil (18.52%); Pb reduced by of 19.07 mg kg^−1^ (3.50%)
Cheng-Kima *et al.* 2016^32^	Metal contaminated soil	*L. rubellus*	Cu, Mn, Pb, Fe, Cr, Ni, Zn, As	90 days	50% reduction in soil amended with mushroom compost
Shameema and Chinnamma (2018)^33^	Metal contaminated soil	Earthworm species not specified	As, Cd, Cr, Ni, Pb, Zn, Hg, Cu	45 days	87–100% reduction in soil metals. Increasing soil metals decreased remediation efficiency

In the work of Marco Parra *et al*. (2010), earthworms (*E. fetida*) and vermicompost were used in the processing and remediation of wastewaters and landfill soils.^30^ Vermicompost was used as an adsorbent substrate for remediation of wastewater contaminated with metals, namely nickel (Ni), chromium (Cr), vanadium (V), and Pb. Earthworms were used for remediation of As and Hg in landfill soils. The earthworms allowed the removal of As and Hg from landfill soils with an efficiency of between 42% and 72% for As, and between 7.5% and 30.02% for Hg. Pattnaik and Reddy (2011) remediated metals, namely Cd, Pb, zinc (Zn), and manganese (Mn), from urban wastes using three species of earthworms.^10^ They observed gradual significant increases (p < 0.05) in metal levels in earthworm tissue from the initial stage to the end. At the end of the 60-day experimental period, Pb and Zn removals of up to 32% and 37%; 45% and 44%; and 51% and 56% were achieved by *E*. *eugeniae*, *E*. *fetida*, and *P*. *excavates*, respectively. In the work of Shahmansouri *et al*. (2005), *E*. *fetida* earthworms sourced from Iran and Australia were used to vermicompost organic wastes, for a period of 2 months.^9^ The metal concentrations in the biosolid decreased with increasing vermicomposting time and this was attributed to the metal-accumulating ability of the earthworms.

Clean Muthia is a vermiremediation project located in Muthia, India.^31^ Due to decades of growth of the chemical and dye sectors, the soil metal and other contaminants levels in the area had substantially increased. The project lasted for two years, divided into two phases, of four quarters each. Industrial waste materials were first excavated from the contaminated site. Thereafter, vermiremediation was carried out on the residual soil with the aid of resident earthworms, a seeded culture of *E*. *fetida* earthworms, earthworm casts mixed with compost, and a microbial solution consisting of photosynthetic bacteria. From the results, soil Cr level decreased from between 192–194 mg kg^−1^ to between 4.5–113.21 mg kg^−1^. Lead showed a decrease from 5,300 mg kg^−1^ to 1,550 mg kg^−1^.

Dada *et al*. (2016) used *L*. *violaceus*, a tropical wetland earthworm, to remediate soils contaminated with Zn, Pb, and Cd *ex situ* in individual and combined concentrations, over a period of 12 weeks.^3^ The presence of *L*. *violaceus* led to significant metal reductions in the contaminated soils. Similarly, Cheng-Kima *et al*. (2016) employed *Lumbricus rubellus* to remediate soil samples contaminated with metals (Cu, Mn, Pb, Fe, Cr, Ni, Zn, As) in Malaysia, for 90 days.^32^ The soil with earthworms and mushroom compost as an amendment had a 50% reduction in soil total metals.

Shameema and Chinnamma (2018) used unnamed species of earthworms to remediate soil contaminated with metals in India.^33^ They recorded significant removal (87–100%) of metals, including As, Cd, Cr, Ni, Pb, Zn, Hg, Cu, at the end of the 14-day experimental period *(Table 3).*

#### Mechanisms of earthworm actions in remediation of inorganic contaminants

**Figure 3:**
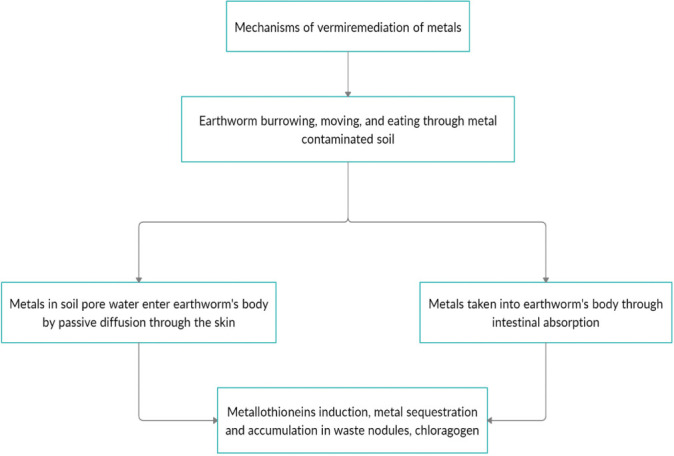
Overview of earthworm mechanisms in metal remediation

Metals are usually not biodegradable; hence, the major known mechanism by which earthworms can remediate metals from the soil environment is through dermal absorption and intestinal intake, and subsequent accumulation into their bodies *(Figure 3).* Many metals are associated with toxicity, and potential plant and animal metal accumulators must possess appropriate mechanisms and devices to cope with high metal burdens.

Earthworms may take in metals and excrete some of these metals through the calciferous glands; un-excreted metals are accumulated in the earthworm's body. The basic mechanism by which earthworms accumulate and cope with a high metal burden is through the induction of metallothioneins, and subsequent sequestration and storage of the metallothionein-bound metals in structures such as waste nodules (brown bodies formed within the body cavity) and chloragogen (fatty cells of the gut wall).^2,10,34^ Metallothionein induction is not only important in vermiremediation of metals, it is crucial to the survival of earthworms in contaminated soil environments.

#### Metallothionein induction, metal sequestration and accumulation in earthworms

The functions of metallothioneins have been described as metal homeostasis and detoxification.^35^ According to Morgan and Morgan (1998),^36^ the major compartmentation site for sequestered metals, including Cd, Pb, Zn, and calcium (Ca), is the chloragosomal matrix, which is a series of intracellular vesicles located around the alimentary canal, especially the posterior part. They indicated that such compartmentation appears to prevent dissemination of large concentrations of these metals into other earthworm tissues and may thus represent a detoxification strategy based on accumulative immobilization. Ireland (1979) also found that Cd and Pb are particularly concentrated in chloragogen cells in *L*. *terrestris* and *Dendrobaena rubida*, where they are sequestered in the form of Cd-metallothioneins and Pb-metallothioneins.^37^

Three Cd-binding metallothionein (MT) isoforms have been discovered in *L*. *terrestris*, wMT1, wMT2, and wMT3.^35,38,39^ Sturzenbaum *et al*. (2001),^38^ in their study of the molecular mechanism underlying bioaccumulation of cadmium in *L*. *terrestris*, concluded that wMT2 is the sole cadmium-responsive MT isoform in earthworms, both at the transcriptional and translational levels. They noted that the protein wMT2 is targeted in intracellular compartments, which are involved in a very efficient detoxification pathway.

Maity *et al*. (2011) evaluated metallothioneins induction as a tool for monitoring metal contamination.^40^ Sexually mature *Lampito mauritii* were exposed to different concentrations of Pb and Zn separately for 28 days and the concentrations of MT were assessed. A significant increase in tissue MT level was recorded in *Lampito mauritii* exposed to Zn- and Pb-contaminated soil. They concluded that MTs are directly involved in metal ion detoxification and help *Lampito mauritii* to survive in metal-contaminated soil, by the sequestration of the toxic metals.

In Nigeria, Dedeke *et al*. (2016) measured the production of metallothioneins in three tropical earthworms (*L*. *violaceous*, *Eudrilus eugeniae*, *Alma millsoni*) collected from three abattoir soils.^41^ Heavy metal [Cu, Zn, Pb, Cd, cobalt (Co), Cr, Ni, Mn] and MTs concentrations were measured in the earthworm tissue and abattoir soil. The concentrations of Cu, Zn, Pb, Cd and Mn were found to be generally higher in abattoir soils and earthworms, relative to the control (undisturbed soil). Metallothioneins concentrations were also higher in earthworms from metal-contaminated abattoir soil. This resulted in significant (p< 0.05) positive correlations between metallothioneins induction and heavy metal concentrations in all the earthworm species. Hence, metallothioneins induction is a major mechanism by which earthworms are able to accumulate, detoxify and sequester high levels of metals in their bodies. The more metals are taken up by the earthworms, the lower the soil metal concentrations.

## Discussion

The reviewed articles and their findings present strong evidence that metallothionein induction is the primary mechanism underlining the process of detoxification, sequestration, and accumulation of metals by earthworms in the metal-contaminated soil environment. Metallothionein induction is a primary survival mechanism in earthworms resident in soil environments with elevated metal levels. Metallothionein induction in earthworms is also useful in monitoring the metal state of a soil environment. However, further studies on mechanisms of vermiremediation of metals are required to confirm whether metallothioneins-induced bioaccumulation and sequestration alone could be responsible for the levels of metal reductions reported in some of the reviewed articles.

### Advantages of vermiremediation technology

Vermiremediation presents a number of advantages, in addition to ridding the soil of contaminants. First, unlike the physicochemical remediation techniques that usually involve soil excavation or treatment with chemicals, vermiremediation is not eco-destructive, but environmentally friendly, and potentially sustainable. Next, where earthworms are employed to remediate soil contaminated with organic wastes, the activities of degrader microorganisms and enzymes are always increased. This eventually leads to improved soil structure and nutrient availability for enhanced plant growth and crop production. In addition, vermiremediation processes, especially those involving organic wastes, bring an added advantage of increasing earthworm biomass that can be harvested and used as livestock feed or for other appropriate purposes. Finally, vermiremediation is a potentially cost-effective remediation technique when compared to some physicochemical remediation methods.^1,42,43^

### Limitations and challenges of vermiremediation

Although vermiremediation is an ideal mechanism to clean up mildly contaminated soil, like other bioremediation methods, it comes with a number of challenges and limitations. First, vermiremediation cannot be used to clean up highly polluted soil. It may only be applied to mildly or, at best, moderately contaminated soils that do not exert overtly toxic effects on earthworms.^8^ Vermiremediation is potentially capable of contaminating the food chain since earthworms serve as food for many birds. However, this risk may be mitigated by employing endogeic earthworms (soil feeding earthworms that live within the mineral layer of soil) that will usually not come out to the soil surface.

In sites where earthworms are used to clean up contaminants, they may not be suitable for use as biomonitoring agents. Spurgeon and Hopkin (2000) expressed the concern that earthworms may develop physiological and genetical adaptations which may have important implications for risk assessments in long-term contaminated sites.^44^ Similar to phytoremediation that utilizes plants to clean up contaminated soil, vermiremediation is potentially a slow process compared to physicochemical remediation techniques.

As in phytoaccumulation (a mechanism of phytoremediation), the fate of the metals taken up and sequestered in the tissues of earthworms generates concern. To mitigate this challenge, we suggest periodic sampling of field earthworms to establish the time of peak metal accumulation. Earthworms can then be harvested, and the accumulated metals can be recovered. Harvesting of earthworms can be done by the use of earthworm skin irritants (e.g. vermifuge or mild soap solution), soil vibration, or by passing a low electric current into the soil; any of these methods will cause the earthworms to come out of their burrows.

Finally, the use of animals for remediation always comes with ethical controversies. However, the use of invertebrates like earthworms should meet with little or no resistance because in many jurisdictions, the term ‘animals’ refers to ‘all live non-human vertebrates'.^45^ More importantly, many vermiremediation processes, especially with organic wastes or contaminants, are harmless to earthworms, improving the soil for their growth and survival.

### Practical field application of earthworms in vermiremediation

Vermiremediation has been practically applied to clean-up contaminated fields as in the case of the Clean Muthia project.^31^ Vermiremediation may be practically applied to clean up contaminated fields using many approaches as highlighted by Hickman and Reid (2008).^8^ Earthworms may be directly seeded to contaminated field or farmland. Earthworms may be co-applied with other organic nutrient media such as cow dung, poultry droppings, or formulated supplements. Organic wastes or substrates may be fed to earthworms with the aim of degrading the wastes or substrates. Vermiremediation may be indirectly deployed by applying vermicompost onto organic contaminant substrates to facilitate their decomposition through the increased activities of degrader microorganisms and enzymes.

Depending on the nature of contaminants and the type of earthworm used in remediation, earthworms may be manipulated to increase intestinal uptake of contaminants, through their feeding habits. Studies have indicated that contaminant bioaccumulation may be enhanced via food limitation.^8,46,47,48^ This suggests that earthworms increase their oral intake of soil particles when driven by hunger stress. Vermiremediation may be deployed as a secondary measure to rid the soil of residual contaminants after applying physicochemical remediation techniques to an overtly polluted soil environment.

## Conclusions

Globally, soil is continually subjected to anthropogenic pollution and contamination from industrial, farming, and other activities. Due to the high costs and environmental degradation associated with traditional physicochemical remediation methods, attention is now shifting to biological *in situ* alternatives. The present review sought to present the state of the literature on the effectiveness of using earthworms to remediate organic and inorganic (metal) soil contaminants. Empirical and theoretical information gathered from the literature showed evidence of earthworms' significant contributions to the mineralization or conversion of organic contaminants, and the reduction of soil inorganic contaminants, mainly metals. Though vermiremediation presents a good long-term biological clean-up option, it can only be applied to mildly contaminated soil. Vermiremediation can be deployed as a secondary measure to rid the soil of residual contaminants after applying physicochemical remediation techniques to an overtly polluted soil environment. However, we recommend more targeted and elaborate studies on the mechanisms of vermiremediation of metals to confirm if metallothionein-induced bioaccumulation and sequestration alone could be responsible for the levels of metal reductions reported in some of the reviewed articles.
